# Supplementary material for: Decision formation in parietal cortex transcends a fixed frame of reference
Source: Neuron. Author manuscript; Available in PMC 2022 Nov 7. (PMC9639165; doi:10.1016/j.neuron.2022.07.019)
Supplement: MMC1 [file NIHMS1833261-supplement-MMC1.pdf]

Neuron, Volume *110*

Supplemental information

Decision formation in parietal cortex transcends a fixed frame of reference

NaYoung So and Michael N. Shadlen

Supplementary figures

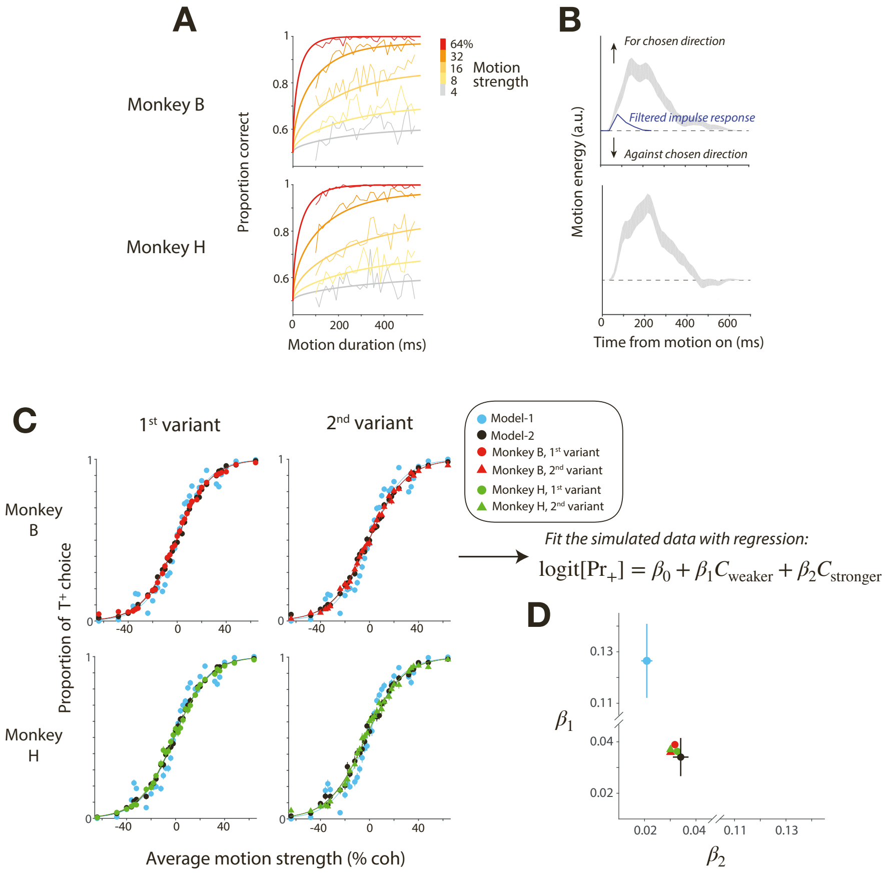

Figure S1. Supplementary behavioral analyses (Related to Figure 1). A–B, Support for evidence accumulation as a function of viewing duration. **A**, Choice accuracy improved as a function of stimulus viewing duration. The proportions of correct trials are shown in thin lines by calculating the running means (15 ms boxcar) of stimulus duration for each motion strength. Thick curves are fits of a bounded drift-diffusion model. The fits suggest that median integration times were 277 ms (monkey B) and 353 ms (monkey H) for the weakest motion strengths. **B**, Psychophysical reverse correlation. The curves show the influence of momentary fluctuations of motion information on the decision in the 0% coherence trials. The sign of the motion energy is positive if it is consistent with the choice. The blue curve shows the time course of an impulse of motion at $t = 0$. The gray traces show the mean $\pm$ s.e.m. Both monkeys use ~ 400 ms of information in the stimulus to form decisions. **C–D**, Both motion pulses affect single decisions. The fact that both pulses influence the decision (Eqs. 4 and 5) does not guarantee that they do so on the same decision. The figure explains the construction of a bivariate statistic that discriminates these possibilities. **C**, Two models are capable of explaining the choice functions from the monkeys. Cyan points are simulations produced by *Model-1*: only one pulse, randomly selected, affects the choice. Black points are simulations produced by *Model-2*: both pulses affect the choice. For both simulations, the weights governing the probability of choosing T^+ are derived from the data (red and green points). Both models are capable of approximating the behavior. **D**, Model-2 is superior. The graph shows the means and standard deviations of $\{\beta_1, \beta_2\}$, the fitted coefficients in a logistic regression that distinguishes the two pulses on the basis of their relative strength (Eq. 5). Under Model-1, the weaker pulse must be more heavily weighted ($\beta_1 > \beta_2$). Model-2 assigns similar weights, as does the fit to data (red & green). Error bars represent $\pm 2\sigma$ based on 1,000 simulated data sets. Each simulation generates 10,000 trials.

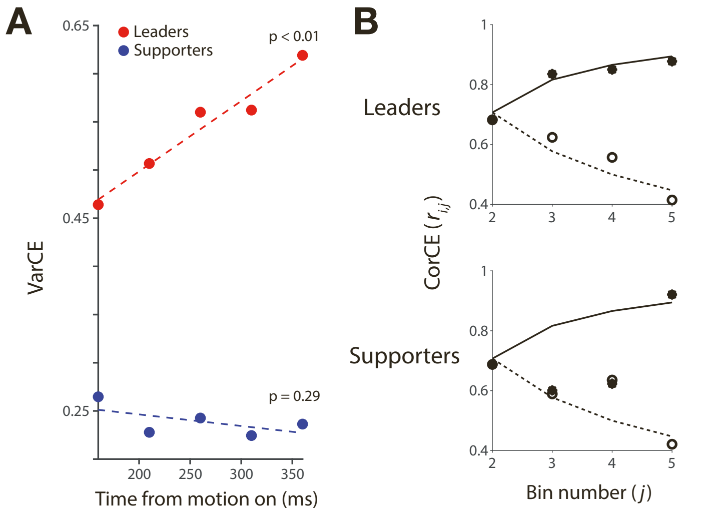

Figure S2. Decision-related neural activity exhibits statistical features consistent with a diffusion-like process (Related to Figure 2). **A**, The variance of the conditional expectation (VarCE) of spike counts is the variance, across trials, of the latent spike rates that gave rise to the spikes. For leader neurons, the VarCE increases linearly during the first 250 ms of putative integration. The linear rise is expected for the accumulation of independent identically distributed samples, as in unbounded diffusion. Supporter neurons do not exhibit this feature. **B**, The CorCE is the pairwise autocorrelation of the latent spike rate at time points i and j. In unbounded diffusion, the correlation is $\rho_{i,j} = \sqrt{i/j}$, captured by a decrease as a function of the separation, $j-i$ (broken lines), and an increase as a function of time for the correlation between neighboring time points, $j-i=1$ (solid lines). Leader neurons approximate this pattern; supporter neurons do not. Symbols are the CorCE estimates from data (open, $r_{1,2\ldots5}$; filled, $\{r_{1,2}, r_{2,3}, r_{3,4}, r_{4,5}\}$). The analysis epoch is the same as in *A*. Bin width is 50 ms, centered at $\{160, 210, 260, 310, 360\}$ ms after motion onset.

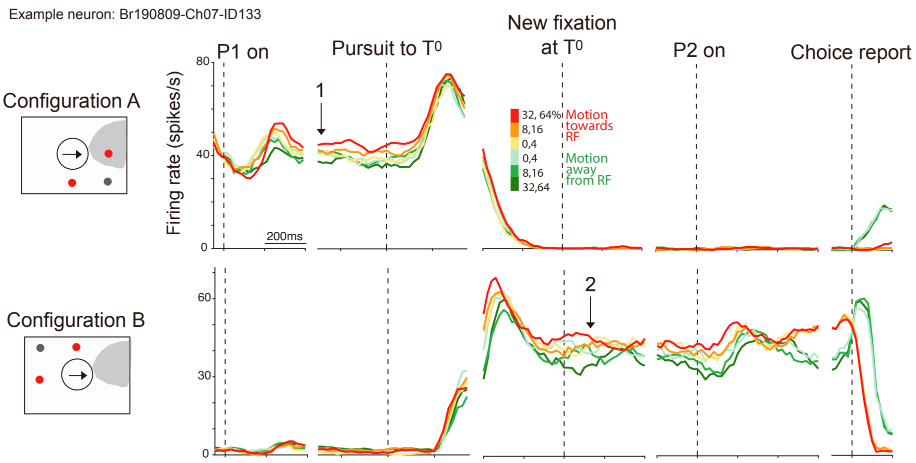

Figure S3. An example neuron that changes its role under different target configurations (Related to Figure 2–3). This neuron was recorded while the monkey performed the 2nd variant of the two-pulse task. In this recording session, two target configurations were randomly interleaved such that one of the choice targets was in the neurons response field when the gaze was at the initial fixation point (Configuration A) or when the gaze was at the new fixation point, T^0 (Configuration B). Accordingly, in Configuration A, this neuron represented the decision variable after P1 (arrow-1), thereby establishing its role as leader. In Configuration B, the neuron played the supporter role. It acquired the decision variable during the pursuit eye movement to T^0 and updated it after P2 (arrow-2). The shading in the diagram is an estimate of the response field, based on the neuron's response during the saccade task (see Methods).

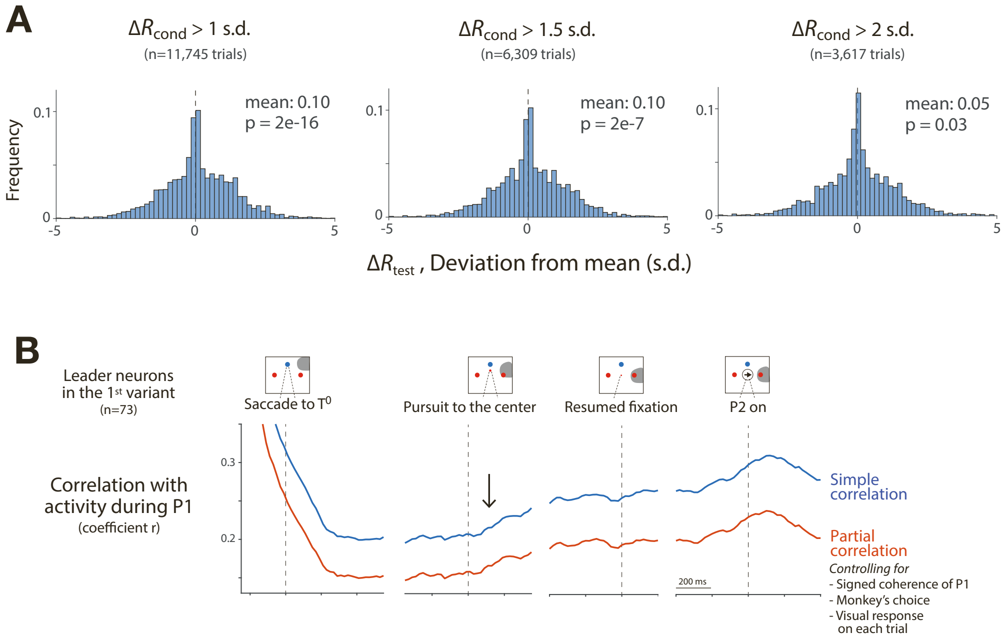

Figure S4. Supplementary analyses on leader neurons during the 1st variant of the two-pulse task (Related to Figure 3). A, Both motion pulses affect the firing rate on single decisions. This analysis uses data from the 1st variant of the two-pulse task, where the same leader neuron responds to both pulses. We assessed the change in firing rate (ΔR) induced by one of the two pulses (termed the *test pulse*) on trials when the other pulse (*conditioning pulse*) is associated with a compelling change in firing rate: $|\Delta R_{\text{cond}}| > z$ s.d., and $\text{sgn}(\Delta R_{\text{cond}})$ is consistent with the choice on the trial. The three histograms show the distributions of ΔR_{test} when z is 1 (*left*), 1.5 (*middle*), and 2 (*right*). ΔR_{cond} and ΔR_{test} are the change in firing rate during 100–400 ms after the onset of the motion pulse. The sign of ΔR_{test} is flipped for the T^- trials, such that the positive ΔR_{test} represents the firing rate change consistent with the monkey's choice. If both pulses affect the neural response, then ΔR_{test} should be positive (i.e., $H_0 : \Delta R_{\text{test}} = 0$; two-tailed t-test). **B**, Autocorrelation of leader neuron activity in the 1st variant of the two-pulse task. The analysis is intended to examine the continuity of the representation of decision-related information across the IEM. Autocorrelation is measured between the response of a single leader in the epoch 200–500 ms after P1 onset and its response later in the trial. Before the IEM, the autocorrelation is high, reflecting the persistent representation of the decision variable. It starts to decrease around the saccade to T^0. As the smooth-pursuit eye movement brings the gaze back to the original fixation point, the autocorrelation increases (*arrow*). The simple autocorrelation (blue) is affected by the visual response to the choice target, the signed coherence of P1, and the choice on the trial. The partial autocorrelation (red) suppresses these factors, leaving only the noise correlation.

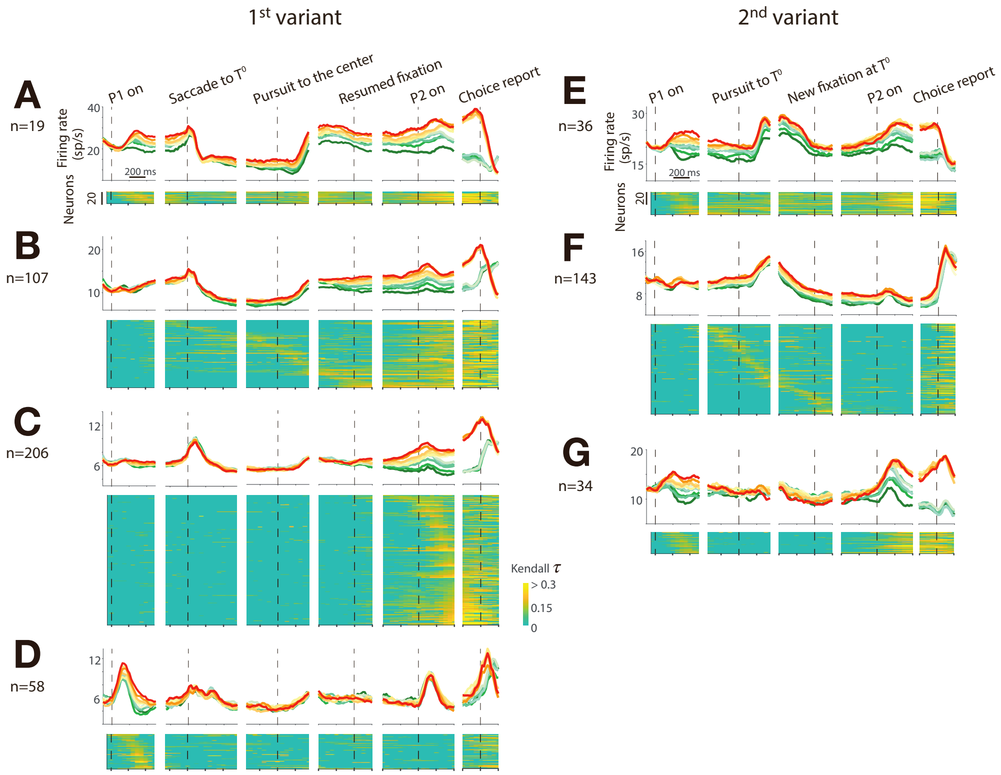

Figure S5. Neurons that are neither leaders nor supporters in the two-pulse tasks (Related to Figure 3). These neurons with large or poorly defined response fields represent the decision variable from various gaze directions. Such neurons cannot associate the decision variable with a particular saccadic choice, but they might play a role in the transfer of the decision variable between leader and supporter neurons. **A–D**, 1st variant. **A**, Neurons with decision-related activity throughout the trial. These have large response fields that contain a choice target viewed from the initial FP and T^0. **B**, Neurons with decision-related activity that begins during the IEM—like supporters—and continues through the P2-viewing epoch. **C**, Neurons that represent the decision variable only after the IEM. These neurons represent the evidence bearing on the final eye movement, consistent with previous studies (Barash et al., 1991; Mazzoni et al., 1996). Alternatively, these neurons may be leader neurons that failed to achieve our criterion for representing the decision variable during the presentation of P1. **D**, Neurons with decision-related activity following P1 but not P2 or the IEM. **E–G**, 2nd variant. **E**, Neurons with decision-related activity throughout the trial. Like the neurons in *A*. **F**, Neurons with decision-related activity only during the IEM. We suspect that the neural response fields are aligned to a choice target only when the gaze is between the initial FP and T^0—that is, during the pursuit eye movement. We lack direct evidence for this, owing to the limited number of target locations in the response field mapping task. **G**, Neurons with decision-related activity following both P1 and P2. Unlike the neurons in *E*, these neurons do not exhibit decision-related activity during the IEM. We suspect that these neural response fields are foveal, where the motion stimulus is displayed (note the short latency response).
